# A rare cause of excruciating chest pain mimicking acute coronary syndrome

**DOI:** 10.1007/s12471-016-0913-8

**Published:** 2016-10-26

**Authors:** L. Hobohm, D. Krompiec, R. Michel, Y. Yang, F. Schmidt, C. Düber, T. Münzel, P. Wenzel

**Affiliations:** 1 Center of Cardiology, Cardiology I, Johannes Gutenberg University Medical Center Mainz, Mainz, Germany; 2 Department of Diagnostic and Interventional Radiology, Johannes Gutenberg University Medical Center Mainz, Mainz, Germany

A 62 year-old male presented to the chest pain unit with chest pain and nausea, reporting that the symptoms occurred one hour after dinner. His medical history included a foudroyant event of pulmonary embolism with embolectomy in 2012.

**Fig. 1:**
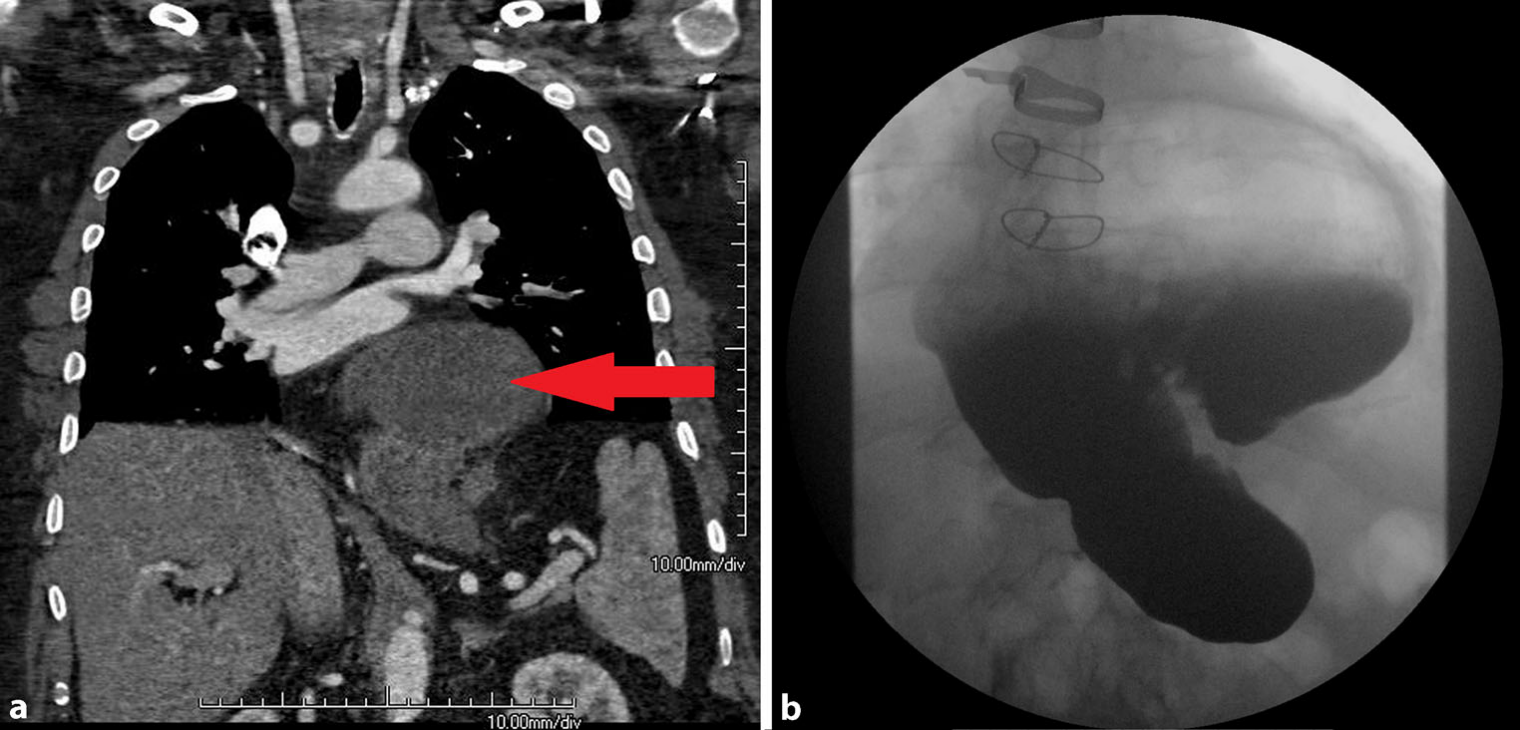
Computed tomography (**a**) and gastrointestinal contrast series (**b**) show a mixed axial and para-oesophageal upside-down stomach (*red arrow*) compressing the left ventricle, without any incarcerated portions of the stomach

Due to clinical deterioration and the history of pulmonary embolism, we decided to perform a contrast computed tomography angiography (CT). We could rule out aortic dissection and pulmonary embolism, However, CT revealed a mixed axial para-oesophageal upside-down stomach (UDS) compressing the left ventricle (Fig. 1).

UDS is the rarest type of hiatal hernia and can manifest clinically in a wide variety of symptoms as demonstrated in this case [1]. As causes of chest pain, gastrointestinal disease other than peptic ulcer or reflux-related diseases which might include UDS were reported to be below 1 % [2]. In UDS patients, complications such as incarceration, volvulus development as well as acute gastric bleeding can lead to a life-threatening emergency with prevalence of 30.4 % and can require immediate surgery [3, 4].
